# Explorative Meta-Analysis of 377 Extant Fungal Genomes Predicted a Total Mycobiome Functionality of 42.4 Million KEGG Functions

**DOI:** 10.3389/fmicb.2020.00143

**Published:** 2020-02-06

**Authors:** Robert Starke, Petr Capek, Daniel Morais, Nico Jehmlich, Petr Baldrian

**Affiliations:** ^1^Laboratory of Environmental Microbiology, Institute of Microbiology of the Czech Academy of Sciences, Prague, Czechia; ^2^Faculty of Science, University of South Bohemia, České Budějovice, Czechia; ^3^Molecular Systems Biology, Helmholtz-Center for Environmental Research-UFZ, Leipzig, Germany

**Keywords:** functional diversity, fungi, microbiome, accumulation curves, modeling

## Abstract

Unveiling the relationship between taxonomy and function of the microbiome is crucial to determine its contribution to ecosystem functioning. However, while there is a considerable amount of information on microbial taxonomic diversity, our understanding of its relationship to functional diversity is still scarce. Here, we used a meta-analysis of completely annotated extant genomes of 377 taxonomically distinct fungal species to predict the total fungal microbiome functionality on Earth with accumulation curves (ACs) of all known functions from the level 3 of KEGG Orthology using both parametric and non-parametric estimates in an explorative data-mining approach. The unsaturated model extrapolating functional diversity as a function of species richness described the ACs significantly better than the saturated model that assumed a limited total number of functions, which suggested the presence of widespread and rare functions. Based on previous estimates of 3.8 million fungal species on Earth, we propagated the unsaturated model to predict a total of 42.4 ± 0.5 million KEGG level 3 functions of which only 0.06% are known today. Our approach not only highlights the presence of widespread and rare functions but points toward the necessity of novel and more sophisticated methods to unveil the entirety of functions to fully understand the involvement of the fungal microbiome in ecosystem functioning.

## Introduction

Ecosystem functioning is mediated by biochemical transformations performed by a community of microbes from every domain of life (Woese et al., 1990). Among them, fungi are globally abundant as microbial saprotrophs, pathogens and mutualists (Peay et al., 2016) and provide a wide range of ecosystem processes such as decomposition of organic carbon (Chapin et al., 2012), deposition of recalcitrant carbon (Six et al., 2006; Schmidt et al., 2011; Clemmensen et al., 2013; Kögel-Knabner, 2017) and transformations of nitrogen and phosphorus (Sinsabaugh et al., 1993; Sinsabaugh, 1994). Thus, their activities may have large-scale consequences for global biogeochemical cycles (Treseder and Lennon, 2015). In every community, multiple organisms from different taxonomic groups can play similar if not identical roles in ecosystem functionality, the so-called functional redundancy (Hubbell, 2005). In fact, functional redundancy of certain functions was shown to be very high with several hundreds to thousands of different taxa expressing the same function within one habitat (Žifčáková et al., 2017). These functions can be statistically inferred based upon homology to experimentally characterized genes and proteins in specific organisms to find orthologs in other organisms present in a given microbiome. This so-called ortholog annotation is performed in KEGG Orthology (Kanehisa et al., 2016a, b) that covers a wide range of functional classes (level 1 of KEGG) comprising cellular processes, environmental information processing, genetic information processing, human diseases, metabolism, organismal system, *brite* hierarchies and functions not included in the annotation of the two databases *pathway* or *brite* (more information about the databases can be obtained under^1^). However, the bottleneck of describing microbiome functions is the low number of fully sequenced and annotated genomes as they are mostly limited to those that have undergone isolation and extensive characterization. Problematically, the vast majority of organisms were not yet studied (Pham and Kim, 2012; Martiny, 2019) and the annotation is based on the similarity to the genomes of the very few studied model organisms. As a consequence, fungal microbiome functionality can be inferred based on the composition of the fungal microbiome and its relation to functional parameters (Starke et al., 2018) as indicated by the frequent use of nuclear ribosomal 18S and ITS2 metabarcoding (5,990 publications with the keyword “18S sequencing” and 2,466 with “ITS2 sequencing” in PubMed as of October 3rd 2019). Although the description of fungal communities is important to assess the drivers of the occurrence and distribution of individual fungal taxa and the composition of their communities, the mere fungal community composition in itself does not provide detailed answers, i.e., about its functionality diversity (Větrovský et al., 2019). Recently, shotgun sequencing of metagenomes (8,857 publications) and metatranscriptomes (514 publications), and mass spectrometric analysis of metaproteomes (426 publications) have gained in popularity as a direct link between taxonomy and function in microbial communities from different environments. Still, our understanding of both functional redundancy and functional diversity and their relationship to taxonomic diversity in these communities is scarce. Here, we use both parametric and non-parametric estimators of functional richness to unveil the relationship between taxonomy and function in fungi with the aim to predict the total fungal microbiome functionality on Earth. For this, we extracted all completely annotated genomes of taxonomically distinct fungal species (*n* = 377) from the Integrated Microbial Genomes and microbiomes (IMG) of the Joint Genome Institute (JGI)^2^ on August 7th 2019 with taxonomic annotation on species level and functional annotation on level 3 of KEGG. Admittedly, the 377 fungal genomes cover only five of 22 fungal phyla (Tedersoo et al., 2018) and 28 of 167 fungal orders (Silar, 2016), indicating the limitation of the small dataset that is heavily biased by taxonomy and geography as not the full fungal tree of life was examined. We analyzed the relationship of gene counts and number of KEGG functions within the fungal kingdom and calculated the parametric estimation comprised of an accumulation curve (AC) (Gotelli and Colwell, 2001) characterized by increasing number of KEGG level 3 functions with increasing species using 1,000 random permutations and its subsequent fit to both a saturated and an unsaturated model. Chao-1 for every 10% of species richness of all 377 fungal species in the database each with 20 replicates represented the non-parametric estimator. We hypothesized limited functionality with a plateau at high species richness and thus a better fit of the saturated model as parametric approach and a stagnating Chao-1 estimator with increasing species richness.

## Materials and Methods

### Metadata Collection of the Total Known Fungal Microbiome Functions

To explore the relationship between diversity and function and to compare genomes across fungal phyla and nutritional guilds, available genomes from fungi (as taxonomic unit) were downloaded from the IMG of the JGI on August 7th 2019. One genome was randomly selected if a species had multiple sequenced genomes to yield taxonomically distinct fungal species. For each genome, the gene counts for each function on the level 3 of KEGG Orthology (Kanehisa et al., 2016a, b) (as functional unit) were retrieved. In total, the database comprised 377 completely annotated fungal genomes with 7,926 KEGG functions (Supplementary Table S1). The sequencing status was denoted as “Draft” for 6, “Permanent Draft” for 339 and “Finished” for 32 fungal genomes. Interspecies redundancy was calculated as the number of KEGG functions covered by one randomly chosen species compared to the total number of functions in all species. Intraspecies redundancy or gene redundancy (Pérez-Pérez et al., 2009) was estimated as average of genes per individual KEGG function in any one species. The nutritional guilds of fungi were annotated on species level or, if not available, at genus level with at least probable confidence ranking using *FUNGuild* (Nguyen et al., 2016). Of all genomes, 330 fungi were assigned to at least one guild. When more than one guild was probable, the annotation was treated as ambiguous and not used for further analysis resulting in the identification of 251 genomes from 17 guilds. If less than three genomes were present in any one guild or any one phylum, the data was removed from the analysis, which was true for the phylum *Blastocladiomycota* (*n* = 2) and the guilds arbuscular mycorrhizal (*n* = 1), epiphyte (*n* = 1), ericoid mycorrhizal (*n* = 1), lichen parasite (*n* = 1), lichenized (*n* = 2), litter saprotroph (*n* = 1), orchid mycorrhizal (*n* = 1), and soil saprotrophs (*n* = 2). The gene counts and KEGG functions per fungal phylum and guild were retrieved as average with standard deviation from the database. To estimate guild and phylum specific differences, both inter- and intraspecies functional redundancy were calculated for every guild and phylum as described for the total database above.

### Accumulation Curves (AC)

Fungal species were randomly added in intervals of one up to the maximum species richness of 377 with 1,000 random permutations per step using the function *specaccum* from the R package *vegan* (Oksanen et al., 2018). The AC of the database permutation was then fitted to a saturated (Equation 1) and an unsaturated model (Equation 2) with the critical point estimated by the term 3*A*_*f*_ as previously described (Čapek et al., 2016). The fit of the models was compared by analysis of variance (ANOVA) and Akaike Information Criterion (AIC) (Bertrand et al., 2006) with a penalty per parameter set to *k* equals two. The total number of KEGG functions in fungi on Earth was predicted using the global species richness estimate of 3.8 million fungi to estimate the potential maximum of KEGG functions even though the estimate is 2.2–3.8 million extant species (Hawksworth and Lücking, 2017) and the Monte Carlo simulation of the function *predictNLS* in the R package *propagate* (Spiess, 2018). To validate the parametric approach, random subsets of the 377 fungal species with different sizes were used to predict the total microbiome functions as described before. In addition, the non-parametric estimation of the lower bound of functional richness was calculated by Chao-1 again using random subsets of the 377 fungal species with different sizes and 20 replicates each (Equation 3).

(1) $$F\,u\,n\,c\,t\,i\,o\,n\,a\,l\,r\,i\,c\,h\,n\,e\,s\,s=\frac{f_{m\,a\,x}*\left[S\,p\,e\,c\,i\,e\,s\,r\,i\,c\,h\,n\,e\,s\,s\right]}{A_{f}+\left[S\,p\,e\,c\,i\,e\,s\,r\,i\,c\,h\,n\,e\,s\,s\right]}$$

(2) $$F\,u\,n\,c\,t\,i\,o\,n\,r\,i\,c\,h\,n\,e\,s\,s=\frac{f_{m\,a\,x}*\left[S\,p\,e\,c\,i\,e\,s\,r\,i\,c\,h\,n\,e\,s\,s\right]}{A_{f}+\left[S\,p\,e\,c\,i\,e\,s\,r\,i\,c\,h\,n\,e\,s\,s\right]}+k*\left[S\,p\,e\,c\,i\,e\,s\,r\,i\,c\,h\,n\,e\,s\,s\right]$$

(3) $$C\,h\,a\,o\,1=F\,u\,n\,c\,t\,i\,o\,n\,a\,l\,r\,i\,c\,h\,n\,e\,s\,s*\frac{a_{1}^{2}}{2\,a_{2}}$$

Here, *f*_*max*_ is the maximum functional richness, *A*_*f*_ the accretion rate of functions with an increasing number of species and *k* the constant of the additive term. Functions found only once or twice are indicated by a_1_ as singletons and a_2_ as doubletons, respectively.

## Results

### Genome Quality and Coverage

The assembled scaffold count was on average 671.1 ± 3,270.9 for 373 genomes as *Meliniomyces bicolor* E, *Podospora anserina* S mat+, *Thielavia appendiculata* CBS 731.68 and *Thielavia arenaria* CBS 508.74 were not found in the database in time of the revision (January 6th 2020). The average scaffold of the fungal genomes indicates high quality of the database but also showed the presence of low quality genomes of *Clarireedia homoeocarpa* with 4,211 scaffolds, of *Neocallimastix* with 13,107 scaffolds, of *Pecoramyces ruminatium* with 16,297 scaffolds, of *Rhizophagus irregularis* with 20,288 scaffolds, of *Moniliophthora perniciosa* with 25,056 scaffolds and of *Tricholoma vaccinum* with 50,239 scaffolds. On average, 24.7 ± 10.2% of the genes in a fungal genome were affiliated a KEGG function.

### Gene Counts and Number of Functions Across Fungal Guilds and Phyla

**FIGURE 1:**
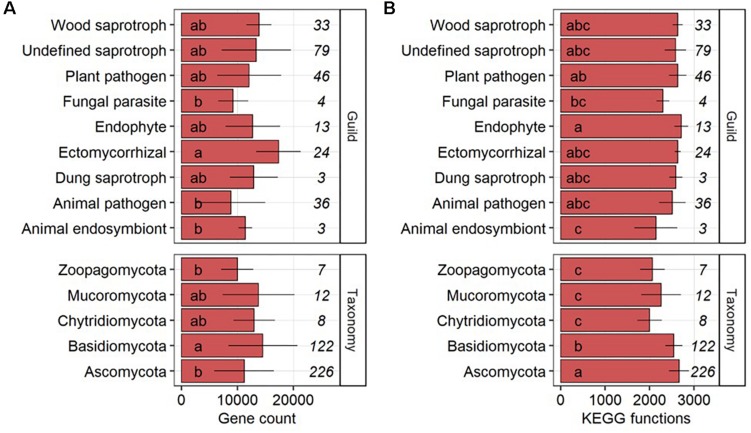
Gene counts **(A)** and the number of different KEGG functions **(B)** per genome across fungal guilds and phyla shown as average with standard deviation. The number of fungal genomes per guild and phylum is given in italic. Groups followed by the same letter are not significantly different according to the HSD-test (*P*-value > 0.05).

The gene count per genome in fungal phyla was significantly (*P*-value < 0.05) higher in *Basidiomycota* as compared to *Ascomycota* and *Zoopagomycota* but *Ascomycota* (*n* = 226) and *Basidiomycota* (*n* = 122) made up 92.3% of the 377 fungal genomes (Figure 1A). On the level of functional ecologies determined by *FUNGuild* (Nguyen et al., 2016), genomes of ectomycorrhizal fungi had on average significantly more genes than animal endosymbionts, animal pathogens and fungal parasites. Undefined saprotrophs were represented by the largest number of fungal genomes (*n* = 79) followed by plant pathogens (*n* = 46), animal pathogens (*n* = 36), wood saprotrophs (*n* = 33), ectomycorrhizal (*n* = 24), endophytes (*n* = 13), fungal parasites (*n* = 4), and animal endosymbionts and dung saprotrophs (both *n* = 3). Significant differences were found in the number of KEGG functions, with more functions in genomes of the *Ascomycota* compared to the *Basidiomycota* that, in turn, had a significantly higher number of functions per genome compared to *Chytridiomycota*, *Mucoromycota*, and *Zoopagomycota* (Figure 1B). On the level of fungal ecologies, genomes of endophytes comprised significantly more KEGG functions than those of animal endosymbionts and fungal parasites.

### Inter- and Intraspecies Functional Redundancy

**FIGURE 2:**
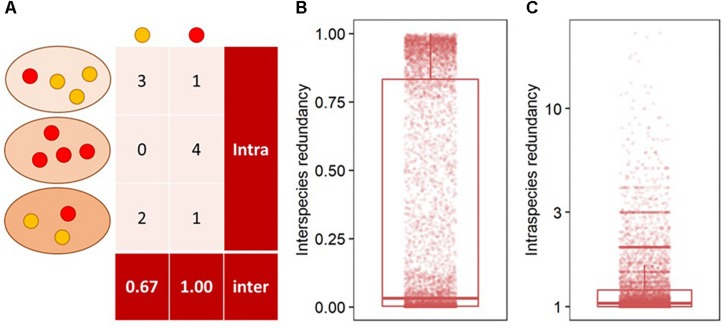
The distribution of interspecies functional redundancy as the total share of functions within fungi relative to the total number of fungal species in the database and intraspecies functional redundancy as the number of replicated KEGG functions within one fungal species in the database. As example, two functions in red and yellow are presented in three taxonomically distinct fungi in different shades of orange **(A)**, and the distribution of interspecies **(B)** and intraspecies functional redundancy **(C)** in all 377 analyzed fungal genomes.

**FIGURE 3:**
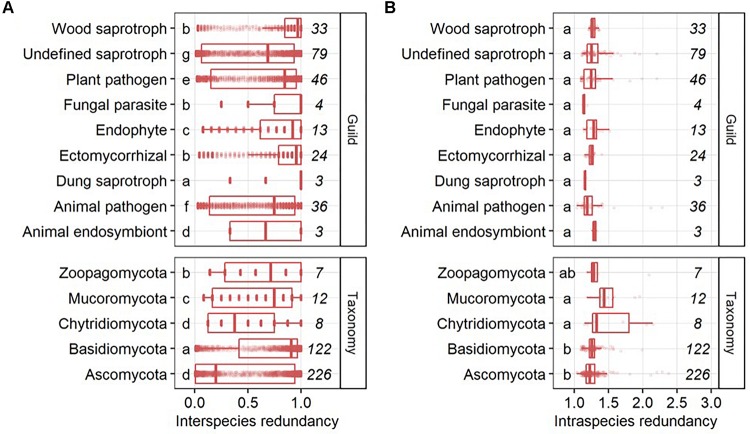
Interspecies **(A)** and intraspecies functional redundancy **(B)** in fungal guilds and phyla. The number of fungal genomes per guild and phylum is given in italic. Groups followed by the same letter are not significantly different according to the HSD test (*P*-value > 0.05).

Interspecies functional redundancy describes the performance of one metabolic function by multiple coexisting and taxonomically distinct organisms (Louca et al., 2016) while intraspecies functional redundancy accounts for the number of replicated functions within one genome (Figure 2A). Across all 377 fungal genomes, the median of interspecies functional redundancy was found to be 0.03 (Figure 2B). Functions showed either high redundancy as 1,592 KEGG functions were found in more than 90% of the fungal genomes or low redundancy with 4,537 KEGG functions in less than 10% of the species. Together, 77.3% of all functions showed either high or low redundancy while 22.7% appeared intermediate with an interspecies functional redundancy between 0.1 and 0.9. The median of intraspecies functional redundancy across all 377 fungal genomes was found to be 2.0 gene copies per KEGG function with a maximum of 118 gene copies (Figure 2C). Among fungal phyla, *Ascomycota* and *Chytridiomycota* showed a significantly lower interspecies functional redundancy than *Mucoromycota* that, in turn, was lower than in *Zoopagomycota* and *Basidiomycota* (Figure 3A). Within fungal guilds, animal pathogens showed a significantly lower interspecies functional redundancy than ectomycorrhizal fungi, fungal parasites and wood saprotrophs that, in turn, were significantly less functionally redundant than animal endosymbionts, plant pathogens, animal pathogens and undefined saprotrophs. Intraspecies functional redundancy was significantly (*P*-value < 0.05) lower in *Ascomycota* and *Basidiomycota* than in *Chytridiomycota* and *Mucoromycota* (Figure 3B). When separated into guilds, intraspecies functional redundancy was not different compared to the mean of all fungal genomes.

### Modeling of the Total Fungal Microbiome Diversity/Functionality Relationship

**FIGURE 4:**
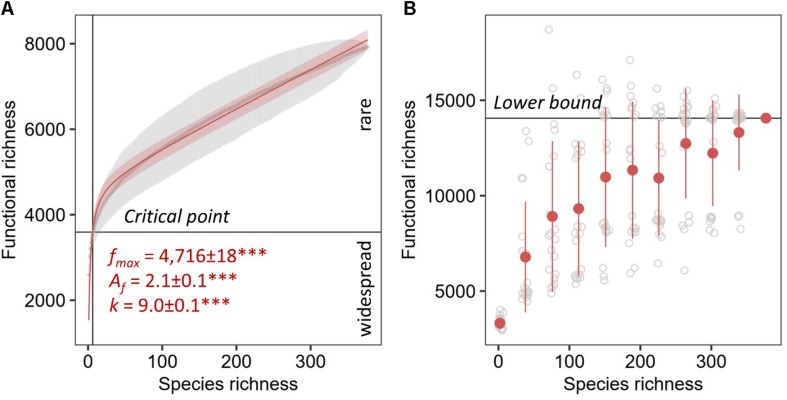
Parametric **(A)** and non-parametric **(B)** estimation of total functional richness. The unsaturated model of the ACs as gray points with error bars for the total known fungal microbiome functions derived from the KEGG database by 1,000 random permutations for every one species richness with 95% confidence intervals. The maximum functional richness is represented by *f*_*max*_, *A*_*f*_ is the accretion rate of functions with increasing number of species, and *k* is the constant of the additive term. Significance of the parameter estimates are indicated by asterisks (^∗∗∗^ equals P < 0.001). The Chao-1 was calculated using 20 replicates shown in gray for every 10% of the total fungal species richness in the database starting with two species.

The unsaturated model described the dependence of functional categories on species richness significantly better than the saturated model with both lower AIC and residual sum of squares (Table 1). The unsaturated model was described by the maximum functional richness *f*_*max*_ of 4,716 ± 18^∗∗∗^ across the 377 fungal species with an accretion rate *A*_*f*_ of 2.1 ± 0.1^∗∗∗^ per fungal species, consistent with the estimate of intraspecies functional redundancy (Figure 4A). However, the relationship did not plateau as indicated by the constant of the additive term *k* that is 9.0 ± 0.1^∗∗∗^. Considering one of the recent fungal species richness estimates of 3.8 million on Earth (Hawksworth and Lücking, 2017) and assuming that the yet unknown fungal microbiome functionality are rare functions, the propagation of the unsaturated model predicted the total fungal microbiome functionality on Earth to be 42,373,186 ± 459,560 KEGG functions (with 41,574,275–43,376,938 as 95% confidence intervals). This estimate was validated by using random subsets of 10, 20, 30, 40, 50, 60, 70, 80, and 90% of all 377 fungal species, which yielded to a plateau of predicted functions when at least 70% of the species were used (Table 2). The non-parametric estimator of functional richness Chao-1 that assumes the existence of a maximum functional richness showed no plateau with increasing species richness of the lower bound estimate (Figure 4B).

**TABLE 1:** The fit of the saturated (S) and the unsaturated model (US) of the accumulation curve (AC) indicated by the Akaike’s An Information Criterion (AIC) and residual sum of squares (Res. Sum Sq), the *P*-value that describes the significant difference between the saturated and the unsaturated model, and the mean prediction with standard deviation (SD) and 95% confidence intervals (CI) at 3.8 million fungal species.

**Model**	**AIC**	**Res. Sum Sq**	***P*-value**	**Prediction**	**SD**	**Lower CI**	**Higher CI**
S	5,843.751	1.17E + 08		7,659	62	7,537	7,779
US	4,693.567	5,514,694	<2.2E-16	42,373,186	459,560	41,574,275	43,376,938

**TABLE 2:** The mean prediction of total fungal microbiome functionality with standard deviation (SD) and 95% confidence intervals (CI) given a richness of 3.8 million fungal species on Earth estimated by the Monte Carlo simulation when random subsamples of the 377 fungal species are used.

**Species (%)**	**Prediction**	**SD**	**Lower CI**	**Higher CI**
377 (100)	42,373,186	459,560	41,574,275	43,376,938
339 (90)	37,575,880	658,902	36,285,040	38,868,781
302 (80)	46,712,785	732,903	45,274,956	48,152,062
264 (70)	38,412,964	315,475	37,794,156	39,031,118
226 (60)	62,515,150	2,085,421	58,421,494	66,607,622
189 (50)	63,362,037	1,645,403	60,134,332	66,591,118
151 (40)	77,516,964	1,427,367	74,713,805	80,321,171
113 (30)	97,942,669	7,026,524	84,131,304	111,757,847
76 (20)	104,882,008	11,188,137	82,875,937	126,831,401
38 (10)	107,435,329	3,650675	100,235,159	114,636,999

*The prediction stabilized at around 40 million functions with random subsets of at least 264 fungal species.*

## Discussion

### Fungal Genomes From IMG

Only 8.5% of the 377 completely annotated fungal genomes were indeed labeled as finished while the majority of the fungal genomes are drafts or permanent drafts, indicating the incompleteness of the fungal genomes. In addition to that, four of the genomes used in the analysis in August 2019 were not found in the database in January 2020, showing the fast turnover of genomes that makes the reanalysis of the data inevitable. Only five of the 377 fungal genomes showed low quality with thousands of scaffolds while the majority of the genomes were of high quality with scaffolds as low as 4 for the finished genomes of *Komagataella pastoris* and *Schizosaccharomyces pombe*. Logically, since the scaffolds of the finished genomes were a magnitude smaller than the ones of the drafts, the completion of fungal genomes is of outmost importance for meta-analyses. Noteworthy, even though finished, the scaffold count of the genome of *M. perniciosa* still indicates low quality which is why not only the completion but also quality control becomes necessary.

### Genome Content

Fungal genomes are small compared to the genome sizes of animals and plants (Tunlid and Talbot, 2002). The higher number of predicted gene sequences in *Basidiomycota* as compared to *Ascomycota* was consistent with the previous examination of 172 fungal species from the same database in 2015 (Mohanta and Bae, 2015). Generally, the genome size of an organism depends on its developmental and ecological needs (Petrov, 2001). A large genome directly increases the nuclear and cellular volumes (Cavalier-Smith, 1978), which helps to buffer fluctuations in concentrations of regulatory proteins or to protect coding DNA from spontaneous mutation (Vinogradov, 1998). Hence, variation in genome size is due to adaptive needs or due to natural selection in different organisms (Petrov, 2001); the so-called adaptive theory of genome evolution. A higher evolutionary rate in *Ascomycota* compared to *Basidiomycota* (Wang et al., 2010) could be directly related to its smaller genome. However, *Ascomycota* were functionally more diverse than *Basidiomycota*. The difference between the number of predicted gene sequences and KEGG functions per genome could also derive from functions without biochemical transformations as those are not captured by KEGG Orthology, i.e., structural proteins necessary to form unique structures found in many fungal guilds. This is clearly a bottleneck to using KEGG for functionality especially in complex multicellular organisms. The potential difference in functional diversity between *Ascomycota* and *Basidiomycota* could be described by different tools for functional annotations such as the carbohydrate-active enzymes (CAZymes). Fungal phyla can comprise of a variety of different trophic modes and thus ecological evaluation of genome content and functional diversity can partly reflect different lifestyles among guilds. In fact, *Ascomycota* contained a total of 12 different guilds compared to 10 guilds in *Basidiomycota*. The larger genome of ectomycorrhizal fungi in comparison to the specialist fungi of animal endosymbionts, animal pathogens and fungal parasites may explain their persistence over competitors such as wood saprotrophic fungi (Tedersoo et al., 2010). Similarly, functional diversity was the highest in endophytic fungi compared to the same specialists as before – animal endosymbionts and fungal parasites. Fungal symbionts such as mycorrhizal or endophytic fungi can have profound effects on plant ecology, fitness, and evolution (Brundrett, 2007) by shaping the plant communities (Clay and Holah, 1999) and their microbiome (Omacini et al., 2001). Plants have been associated with endophytic (Krings et al., 2007) and mycorrhizal fungi (Redecker et al., 2000) for more than 400 million years but unlike mycorrhizal fungi that colonize plant roots and grow into the rhizosphere, endophytes reside entirely within plant tissues and can grow within roots, stems and leaves (Sherwood and Carroll, 1974; Carroll, 1988; Mueller et al., 2004). Endophytes comprise of a higher functional arsenal by aiding to the health and survival of the host plant acting toward biotic threats such as pathogens or herbivores but also environmental factors such as heat or water stress, and soil factors such as nutrient availability or salinity (Hardoim et al., 2015). Here, we demonstrate that the difference in lifestyle between endophytic and ectomycorrhizal fungi could derive from functional diversity and genome content, respectively, despite both being associated with plants. Admittedly, the importance of the genome content of mycorrhizal fungi is questionable as the convergent evolution of the mycorrhizal habit occurred via the repeated evolution from saprotrophic ancestors (Kohler et al., 2015) but genomic sequencing revealed substantial variation between species from the same functional guild resulting in the analysis of trait-based approaches for understanding fungal activity instead of continuous classifications (Zanne et al., 2019). A trait-based approach to measure enzyme activity directly will be necessary when taxa do not fit into functional guilds (Peay et al., 2016). Indeed, some endophytic fungi of roots, leaves and stems have recently shown to protect plants from pathogens (Arnold et al., 2003), herbivores (Clay et al., 2005), and challenging environmental conditions (Márquez et al., 2007) while others might facilitate disease in the presence of more virulent pathogens (Busby et al., 2013). Hence, the genome content and its functional diversity may relate better to the trait-based approach.

### Functional Redundancy

Most major biogeochemical reactions are driven by a limited set of metabolic pathways that are found in a variety of microbial clades (Falkowski et al., 2008). In line with this observation, taxonomic diversity was found to correlate strongly with functional diversity and many ectomycorrhizal fungal species with similar ecological effects co-occurred in the same community (Rineau and Courty, 2011), implying a high interspecies functional redundancy to mobilize nutrients from organic compounds (Walker et al., 1999; Bolger, 2001). Here, interspecies redundancy was either high or low for 77.3% of all KEGG functions. Hence, functions appear to diverge into two groups: (i) highly redundant across fungal species (in more than 90% of the species) or (ii) unique to only a few (in less than 10% of the species). However, the presence of intermediate functions found in between 10 and 90% of the species suggests a less strict classification with more than two groups. Generally, low intraspecies functional redundancy could derive from different KEGG functions performing functionally similar processes. In fact, all malate dehydrogenases perform the same metabolic function but are annotated by different KEGG functions (K00024-K00029) due to their involvement in a variety of metabolic pathways. The highest intraspecies redundancy was found for the ascomycete *Coccidioides immitis* that featured 35 gene copies for the prolyl 4-hydroxylase (K00472, EC 1.14.11.2) and 29 gene copies for the glutathione S-transferase (K00799, EC 2.5.1.18); both are functions with low interspecies redundancy. Noteworthy, the median intraspecies redundancy of *C. immitis* of 1.0 was lower compared to the other fungi in the database, which could relate to a rather uncommon lifestyle and a higher share of unique but not essential functions. Indeed, comparative genomic analysis revealed that *C. immitis* is a primary pathogen of immunocompetent mammals (Sharpton et al., 2009). Functions with high interspecies and high intraspecies redundancy included the yeast amino acid transporter (K16261) with 14.5 ± 8.2 gene copies found in 351 of the 377 fungal species (93.1%), the salicylate hydroxylase (K00480, EC 1.14.13.1) with 13.9 ± 9.5 gene copies (81.7%) and the glutathione S-transferase (K00799, EC 2.5.1.18) with 12.7 ± 11.7 gene copies (98.9%). All of the above belong to the maintenance apparatus of the fungus, namely the transport of amino acids, the incorporation/reduction of oxygen by salicylate hydroxylase, and the detoxification of xenobiotic substrates by glutathione S-transferase. Hence, functions with high interspecies and high intraspecies redundancy are both widespread and essential to every fungus. Functions with high interspecies and low intraspecies redundancy were not found in the 377 genomes. Logically, there might not exist widespread functions that are not essential. As suggested by the interspecies redundancy, the better fit of the unsaturated model inferred the presence of two types of microbiome functions. On the one hand, widespread functions rapidly increase with the number of species and are ubiquitously abundant in all living fungi. In total, nine functions were found in all of the 377 fungal species. All of these are crucial to sustain life – examples include the ribose-phosphate pyrophosphokinase (K00948, EC 2.7.6.1) necessary for nucleotide synthesis and the citrate synthase (K01647, EC 2.3.3.1) of the TCA cycle or the superoxide dismutase (K045654, EC 1.15.1.1) that is an important antioxidant defense mechanism. The number of widespread functions is likely to be limited and amounted to 3,593 ± 31 KEGG functions (with 3,534–3,654 as 95% confidence intervals); nearly half of all known functions as of today. Otherwise, roughly 4,300 functions are rare and increase at a much slower rate with an increasing number of species but require time and the evolution of “dead ends,” i.e., species that were unable to evolve a particular function. The addition of more fungal genomes may increase the interspecies redundancy but it is questionable if a function only found in a few of the 377 fungal species can potentially be widespread amongst fungi.

### The Total Fungal Microbiome Functionality

The propagation of the unsaturated model describing the increase in functions for 377 fungal species to 3.8 million fungi on Earth as the potential maximum of functional diversity (Hawksworth and Lücking, 2017) yielded 42.4 ± 0.5 million KEGG functions, which plateaued at around 40 million functions when a random subset of at least 264 species (70%) were used to compute the AC, fit the unsaturated model and propagate to the global species richness on Earth. Logically, this suggests that the addition of new species will not result in a different prediction of total functionality. However, the addition may lead to a different categorization into widespread and rare functions of individual functions and can thus result in a different prediction. Further support for the unsaturated model was the non-parametric estimator of functional richness. Chao-1 provides a lower bound estimate for the functional richness (Chao, 1984, 1989), assuming that the number of functions plateaus with higher number of species. In our data, however, the Chao-1 estimator did not plateau with an increasing number of randomly chosen fungal species. In theory, more and more species must be sampled until no new functions are found and the AC reaches an asymptote (Gotelli and Colwell, 2011) but, in practice, this approach is generally impossible due to the prohibitively large number of species that need to be sampled to reach an asymptote (Chao et al., 2009). Indeed, every of the predicted 3.8 million fungal species on Earth must be sampled in order to reach the maximum of 42.4 ± 0.5 million KEGG functions. Admittedly, the exploration beyond the limits of the data is likely to be imprecise and the predictions of fungal microbiome functionality may differ once additional fungal genomes with potentially new functions have been added. It is also likely that the discovery rate of novel functions decreases to a saturated model with higher species richness, but the validation requires both more sequenced genomes of taxonomically distinct fungi and the discovery of novel functions. As of today, our understanding of fungal microbiome functionality is likely limited to a marginal part of all functions.

Taken together, we suggest the presence of widespread and rare functions within the fungal microbiome. Our predictions revealed a potential for millions of as-yet unknown functions that, logically, can only be unveiled by novel and more sophisticated methods. However, due to the vast amount of yet unknown functions, it is questionable (i) if the relationship between taxonomy and function is in fact explained by an unsaturated model, (ii) if only two types of functions exist as the distribution of interspecies functional redundancy pointed toward the presence of intermediate functions, (iii) if it is similar when different tools for the functional annotation are used and (iv) if the predictions change once more fungal genomes have been sequenced, finished and completely annotated. Similarly, the total bacterial microbiome functionality could be predicted using more than 70,000 completely annotated genomes of bacterial species. Given the predictions of 100 million bacterial species on Earth (Curtis et al., 2002; Schloss and Handelsman, 2006) together their lifestyle as micro-environment niche specialists (Simard et al., 2012), the total bacterial microbiome functionality is likely much higher as recently shown by proteins (Starke et al., 2019a) and hence, our understanding of the involvement of microbes in ecosystem functioning even lower. Noteworthy, on average only a quarter of the genes in the fungal genomes were affiliated with a KEGG function, clearly demonstrating the limitations of the database as the prediction of functionality technically excluded three quarter of the full functional potential. However, it is likely that other phylogenomic databases provide similar coverages which is why different approaches and definitions of functionality will be necessary to estimate the actual number of functions.

## Data Availability Statement

Publicly available datasets were analyzed in this study. This data can be found here: https://img.jgi.doe.gov.

## Author Contributions

RS designed the study, performed the computational analysis, modeled the data, and wrote the manuscript. All authors reviewed and approved the final version of the manuscript.

## Conflict of Interest

The authors declare that the research was conducted in the absence of any commercial or financial relationships that could be construed as a potential conflict of interest.

## Footnotes

1 https://www.genome.jp/kegg/kegg3.html

2 https://img.jgi.doe.gov/
